# Progressive cone and cone-rod dystrophies: clinical features, molecular genetics and prospects for therapy

**DOI:** 10.1136/bjophthalmol-2018-313278

**Published:** 2019-01-24

**Authors:** Jasdeep S Gill, Michalis Georgiou, Angelos Kalitzeos, Anthony T Moore, Michel Michaelides

**Affiliations:** 1 UCL Institute of Ophthalmology, University College London, London, UK; 2 Moorfields Eye Hospital NHS Foundation Trust, London, UK; 3 Ophthalmology Department, University of California San Francisco School of Medicine, San Francisco, California, USA

**Keywords:** dystrophy, genetics, imaging, retina

## Abstract

Progressive cone and cone-rod dystrophies are a clinically and genetically heterogeneous group of inherited retinal diseases characterised by cone photoreceptor degeneration, which may be followed by subsequent rod photoreceptor loss. These disorders typically present with progressive loss of central vision, colour vision disturbance and photophobia. Considerable progress has been made in elucidating the molecular genetics and genotype–phenotype correlations associated with these dystrophies, with mutations in at least 30 genes implicated in this group of disorders. We discuss the genetics, and clinical, psychophysical, electrophysiological and retinal imaging characteristics of cone and cone-rod dystrophies, focusing particularly on four of the most common disease-associated genes: *GUCA1A*, *PRPH2*, *ABCA4* and *RPGR*. Additionally, we briefly review the current management of these disorders and the prospects for novel therapies.

## Introduction

Inherited retinal diseases (IRDs) are a large group of clinically and genetically heterogeneous conditions which constitute the leading cause of legal blindness in England and Wales among working-age adults, and the second most common in childhood.^1^ One subgroup of IRDs is the progressive cone dystrophies (CODs) and cone-rod dystrophies (CORDs), characterised by the primary degeneration of cone photoreceptors often with later rod involvement. Their estimated incidence ranges from 1 in 20 000–100 000.^2 3^

Inherited disorders of cone function are classically divided into two subtypes: stationary^4^ and progressive.^5^ The stationary cone disorders (cone dysfunction syndromes) are congenital/early-infantile onset and give rise to purely cone dysfunction, whereas progressive cone dystrophies are of later onset and usually also involve rod photoreceptors. There may, however, be overlap as some forms of cone dysfunction syndrome, such as achromatopsia,^4 6 7^ are associated with limited progression over time in a minority of subjects.

Recent advances in molecular genetics, particularly next-generation sequencing (NGS), have greatly improved molecular diagnosis, as the underlying causative genes and mutations can be identified in a large proportion of patients with COD and CORD. Many of these genes encode proteins involved in photoreceptor structure, or the phototransduction cascade.

## Photoreception and the phototransduction cascade

Rod photoreceptors contain rhodopsin photopigment, whereas cone photoreceptors contain one of three types of opsin: S-cone, M-cone or L-cone opsin. Disease-causing sequence variants in the genes encoding the latter two cone opsins (*OPN1MW* and *OPN1LW*, respectively) are implicated in X linked (XL) Bornholm eye disease and S-cone monochromatism.^8^ The latter disorder, although usually stationary, may show a progressive phenotype.

### Photoreceptor activation

**Figure 1:**
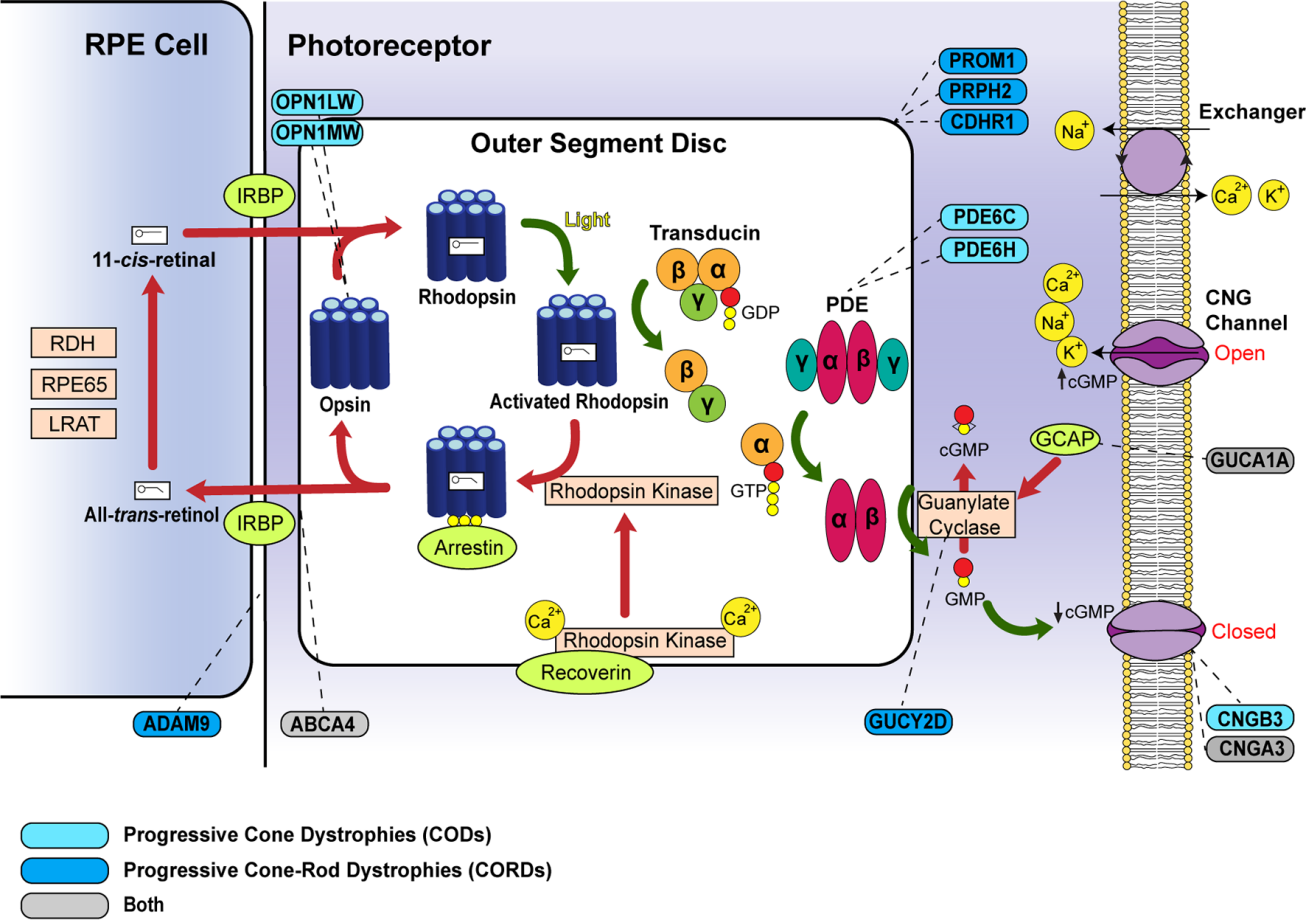
Schematic diagram of the phototransduction cascade including genes related to progressive cone dystrophies (CODs) and cone-rod dystrophies (CORDs). The cascade is triggered in the photoreceptor disc membrane by light-induced activation of rhodopsin, which subsequently activates transducin and phosphodiesterase (PDE). Activated PDE leads to cGMP hydrolysis to GMP. The decreased intracellular cGMP levels induce cation channel closure in the outer segment membrane and result in photoreceptor hyperpolarisation. Steps leading to photoreceptor activation are denoted by *green arrows*, whereas those causing photoreceptor deactivation are marked by *red arrows*. Corresponding genes for the proteins associated with CODs/CORDs are indicated by *dashed lines*. COD-associated genes are coloured in *light blue* and CORD-associated genes in *dark blue*, while those that can cause either phenotype are in *grey*. cGMP, cyclic GMP; CNG, cyclic nucleotide-gated; GCAP, guanylate cyclase-activating protein; GDP, guanosine diphosphate; GMP, guanosine monophosphate; GTP, guanosine triphosphate; IRBP, interphotoreceptor retinoid-binding protein; LRAT, lecithin retinol acyltransferase; PDE, phosphodiesterase; RDH, retinol dehydrogenase; RPE65, retinal pigment epithelium 65 kDa.

The first stage of phototransduction involves the light-induced activation of rhodopsin, in which its bound chromophore, 11-*cis*-retinal, is isomerised into all-*trans*-retinol (figure 1).^9^ The resulting conformational change allows rhodopsin to interact with transducin, a guanine nucleotide-binding protein, to trigger dissociation of its α-subunit. In turn, the transducin α-subunit activates cyclic guanosine monophosphate (cGMP)-phosphodiesterase (PDE) by removal of its inhibitory γ-subunits, thus reducing intracellular cGMP levels and inducing closure of cGMP-gated (CNG) cation channels. As the membrane Na^+^-Ca^2+^-K^+^ exchanger channels remain active with ongoing ion exchange, the CNG channel closure leads to decreased intracellular cation levels and cell hyperpolarisation.^10^

Recessive variants in *PDE6C* and *PDE6H*, which encode the cone photoreceptor PDE α-subunits and γ-subunits, respectively, are associated with autosomal recessive (AR)-COD.^11 12^ Similarly, disease-causing variants in *CNGA3* and *CNGB3*, which encode the CNG channel α-subunit and ß-subunit, respectively, impair the CNG-mediated dark current that is normally modulated by light inputs. These sequence variants typically result in achromatopsia, although some variants may cause an AR-COD or CORD phenotype.^6 7 13^

### Photoreceptor deactivation

Following their activation, the phototransduction molecules enter a refractory period during which intracellular mechanisms return the photoreceptor to its dark state. The activated photopigment (rhodopsin) is phosphorylated by a G-protein-coupled receptor kinase (rhodopsin kinase), after which it is preferentially bound and inactivated by arrestin. Another mechanism by which the photoreceptor returns to its basal state is via retinal guanylate cyclase (RetGC1, encoded by *GUCY2D*). This enzyme replenishes intracellular cGMP levels following activation by Ca^2+^-sensitive guanylate cyclase-activating proteins (GCAPs). The interaction of intracellular cGMP with membrane CNG channels mediates an open configuration in the latter, causing cation influx and membrane depolarisation.^9^

Autosomal dominant (AD)-COD and CORD phenotypes may be associated with variants in *GUCA1A*, which encodes the GCAP1 protein. The common p.(Tyr99Cys) substitution is most associated with COD,^14^ but also with a range of other phenotypes,^15^ while the p.(Pro50Leu) variant often results in CORD.^16^ In contrast, *GUCY2D* variants are arguably associated with less phenotypic variability and result in AD-CORD.^17^ The degree of rod involvement is, however, milder in *GUCY2D* single-variant families compared to those with complex sequence variants.^18 19^

## Genetic and clinical characteristics of COD and CORD

### Molecular pathology

To date, mutations in 32 genes are reported to cause COD or CORD (table 1). There are currently 6 identified genes that predominantly cause COD and 22 that lead to CORD. However, there is considerable overlap with the majority of genes associated with rod involvement over time.

**Table 1:** Summary of the identified disease-causing genes in progressive cone and cone-rod dystrophy

Classification and inheritance pattern	Gene abbreviation	Gene name	Gene locus	Potential function	Other associated phenotypes (OMIM)
**Progressive** **c** **one** **d** **ystrophy** **(COD)**					
Autosomal recessive	CACNA2D4	Voltage-dependent calcium channel alpha-2/delta-4	12p13.33	Neurotransmitter release	–
	CNGB3	Cyclic nucleotide-gated channel beta-3	8q21.3	Phototransduction	ACHM
	PDE6C	Cone-specific phosphodiesterase alpha subunit	10q23.33	Phototransduction	ACHM
	PDE6H	Cone-specific phosphodiesterase gamma subunit	12p12.3	Phototransduction	ACHM
X linked	OPN1LW	Long-wave-sensitive opsin 1	Xq28	Phototransduction	BCM, BED
	OPN1MW	Medium-wave-sensitive opsin 1	Xq28	Phototransduction	BCM, BED
**Progressive cone-rod dystrophy (CORD)**					
Autosomal dominant	AIPL1	Aryl-hydrocarbon-interacting protein-like 1	17p13.2	Tissue development	LCA, RP
	CRX	Cone-rod homeobox-containing gene	19q13.33	Tissue development	LCA, MD
	GUCY2D	Guanylate cyclase 2D	17p13.1	Photoreceptor recovery	CACD, LCA
	PITPNM3	Membrane-associated phosphatidylinositol transfer protein 3	17p13.2-p13.1	Tyrosine kinase signalling	–
	PROM1	Prominin 1	4p15.32	Outer segment morphogenesis	MD, RP
	PRPH2	Peripherin 2	6p21.1	Outer segment morphogenesis	CACD, LCA, MD, RP
	RAX2	Retina and anterior neural fold homeobox 2	19p13.3	Tissue development	–
	RIMS1	Protein regulating synaptic membrane exocytosis 1	6q13	Neurotransmitter release	RP
	UNC119	Human homologue of *C.elegans* UNC119 protein	17q11.2	Neurotransmitter release	–
Autosomal recessive	ADAM9	A disintegrin and metalloproteinase domain 9	8p11.22	Outer segment–RPE junction	–
	C21ORF2	Chromosome 21 open reading frame 2	21q22.3	Ciliogenesis	–
	C8ORF37	Chromosome eight open reading frame 37	8q22.1	Unknown	RP
	CDHR1	Cadherin-related family member 1	10q23.1	Outer segment morphogenesis	RP
	CEP78	Centrosomal protein 78-kD	9q21.2	Ciliogenesis	–
	CERKL	Ceramide kinase-like	2q31.3	Photoreceptor survival	RP
	KCNV2	Potassium voltage-gated channel subfamily V 2	9p24.2	Unknown	–
	POC1B	Proteome of the centriole 1B	12q21.33	Intraflagellar transport	–
	RAB28	Ras-associated protein 28	4p15.33	Intraflagellar transport	–
	RPGRIP1	Retinitis pigmentosa GTPase regulator protein 1	14q11.2	Intracellular trafficking	LCA
	SEMA4A	Semaphorin 4A	1q22	Tissue development	RP
	TTLL5	Tubulin tyrosine ligase-like family member 5	14q24.3	Steroid receptor signalling	–
X linked	CACNA1F	Voltage-dependent calcium channel alpha-1F	Xp11.23	Neurotransmitter release	AIED, CSNB
**Both**					
Autosomal dominant	GUCA1A	Guanylate cyclase activator 1A	6p21.1	Photoreceptor recovery	–
Autosomal recessive	ABCA4	ATP-binding cassette subfamily A member 4	1p22.1	Retinoid cycle	MD, STGD
	CNGA3	Cyclic nucleotide-gated channel alpha-3	2q11.2	Phototransduction	ACHM
X linked	RPGR	Retinitis pigmentosa GTPase regulator	Xp11.4	Intraflagellar transport	MD, RP

ACHM, achromatopsia; AIED, Aland Island eye disease; BCM, blue cone monochromacy; BED, Bornholm eye disease; CACD, central areolar choroidal dystrophy; CSNB, congenital stationary night blindness; LCA, Leber congenital amaurosis; MD, macular dystrophy; OMIM, online Mendelian inheritance in man; RP, retinitis pigmentosa; RPE, retinal pigment epithelium; STGD, Stargardt disease.

The proteins encoded by these genes perform a diverse range of functions in the photoreceptor, including phototransduction (as outlined above), outer segment (OS) morphogenesis (*CDHR1*, *PROM1*, *PRPH2*),^20–22^ intraflagellar transport (*RAB28*, *RPGR*)^23 24^ and neurotransmitter release (*RIMS1*, *UNC119*).^25 26^

**Figure 2:**
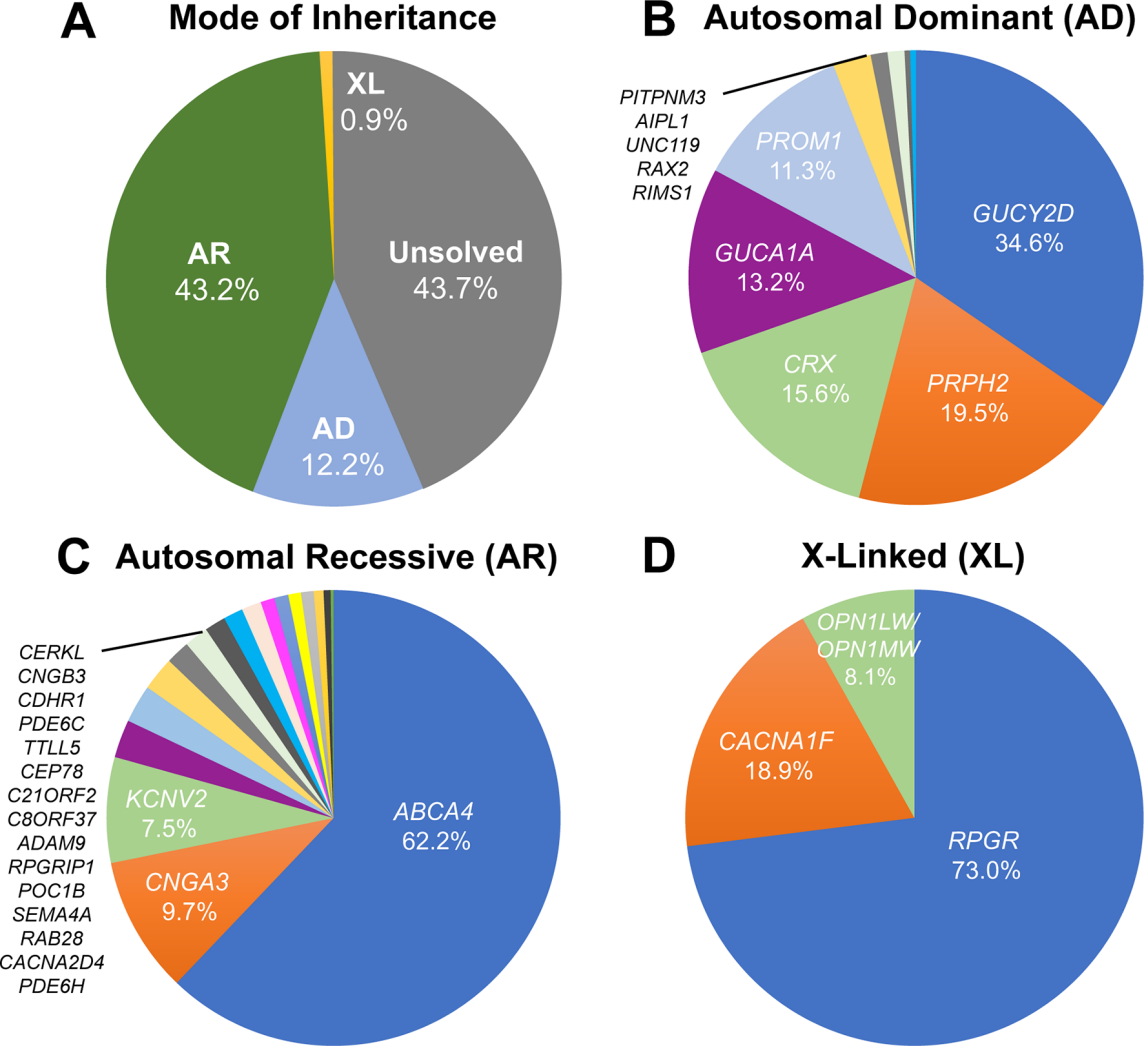
Frequency of disease-causing genetic variants leading to progressive cone dystrophies (CODs) and cone-rod dystrophies (CORDs), using studies with clearly indicated cohort sizes (listed in online supplementary tables 1-3). (A) Prevalence of the mode of inheritance for CODs and CORDs. The underlying disease-causing gene is identified in 56.3% of COD/CORD cases, of which most (43.2%) are of autosomal recessive (AR) inheritance. This is followed by autosomal dominant (AD) inheritance (12.2%) and X linked (XL) inheritance (0.9%) patterns. The remaining 43.7% of patients are unsolved with regard to molecular causation. (B) AD inheritance of CODs and CORDs. Mutations in 10 genes are currently associated with AD-COD/CORD, over 75% of which are accounted for by *GUCY2D*, *PRPH2*, *CRX* and *GUCA1A*. (C) AR inheritance of CODs and CORDs. Mutations in 18 genes are currently associated with AR-COD/CORD, of which *ABCA4* is by far the most common (62.2%). (D) XL inheritance of CODs and CORDs. Mutations in 4 genes are currently associated with XL-COD/CORD, of which *RPGR* accounts for 73.0% of cases.

In order to assess the relative contribution of each gene in each mode of inheritance (AD, AR and XL), all publicly available literature on CODs and CORDs (PubMed search November 2018) in which unrelated probands were genetically investigated was analysed (online supplementary tables 1–3). The number of patients with COD/CORD secondary to disease-causing variants in a given gene was counted as a percentage of the total number of COD/CORD in each cohort. Using this approach, we estimated that the disease-causing gene is identified in 56.3% of patients with COD/CORD, while the remaining 43.7% of cases are unsolved (figure 2A). The majority of molecularly defined disease is recessively inherited, with variants in the *ABCA4* gene accounting for 62.2% of AR-COD/CORD cases. In AD-inherited and XL-inherited cases, variants in *GUCY2D* (34.6%) and *RPGR* (73.0%) constitute the most prevalent monogenic causes of disease, respectively, among currently identified genes (figure 2B–D).

10.1136/bjophthalmol-2018-313278.supp1 Supplementary data

### Clinical presentation

COD presents with loss of central vision, photophobia and colour vision disturbance. Since cone function is usually initially normal, nystagmus is often absent. COD is distinguishable from CORD by the absence of early nyctalopia, which occurs in the latter due to concomitant rod degeneration. However, the majority of patients with COD develop rod dysfunction or loss as the disease progresses.^4^ In most cases, CODs and CORDs affect colour discrimination in all three colour axes due to parallel cone degeneration of the three opsin subtypes. Kellner *et al*^27^ and Went *et al*^28^ present notable exceptions to this with preferential degeneration of L-opsin and S-opsin cones, respectively, thereby causing a protan or tritan defect.

A longitudinal study by Thiadens *et al*^29^ demonstrated earlier symptomatic onset in CORD than COD (12 vs 16 years), as well as a more severe disease course based on psychophysical testing. Visual acuity in more than half of patients with CORD (n=83) deteriorated to legal blindness by the age of 23 years, compared with 48 years in COD (n=98), although there was a large degree of individual variability and overlap.

### Clinical investigation

#### Psychophysical assessment

Reduced visual acuity is the earliest manifestation of COD/CORD, generally occurring in the first decade of life and not significantly improved by spectacle wear.^2^ The presence of an isolated central scotoma on visual field testing is typical in patients with COD, but cannot be used alone for discriminating the diagnosis from CORD. A significant proportion of patients with CORD retain peripheral vision at the time of disease onset, and develop a peripheral scotoma up to 10 years later.^29^ In general, CODs and CORDs lead to marked visual loss at an earlier age than retinitis pigmentosa (RP), a rod-cone dystrophy, and are thus arguably more severe conditions.^2^

#### Electroretinography

The earliest electroretinography (ERG) finding in COD/CORD is a delayed 30 Hz flicker ERG implicit time, which selectively assesses cone response.^2^ This is followed by deterioration of the 30 Hz flicker ERG amplitude, and reduced a-wave and b-wave amplitudes of the single flash photopic ERG. Scotopic function is preserved in early disease, but is usually affected in late disease. In corroboration with psychophysical findings, longitudinal ERG in CORD shows a faster rate of cone functional decline than in patients with COD.^30^

In one disorder, *KCNV2*-associated retinopathy, the ERG findings are characteristic and diagnostic; there is an abnormal cone ERG with ‘supernormal’ rod responses.^31^ Despite its nomenclature, this disorder is not associated with enhanced rod function measured psychophysically.^32^

#### Retinal imaging

Funduscopy in COD classically reveals a bull’s eye maculopathy, but may more often identify bilateral and relatively symmetrical retinal pigment epithelium (RPE) disturbance/atrophy with progression over time.^5^ Peripheral RPE atrophy and bone spicule pigmentation are observed in advanced stages of CORD, whereas the retinal periphery is typically normal in CODs due to rod preservation. White flecks have, however, been described at the level of the RPE,^33^ with such cases likely to develop rod dysfunction over time. Other reported findings include a dark choroid sign on fluorescein angiography^34^ and a tapetal-like sheen in XL-CORD.^35^

Fundus autofluorescence (FAF) and optical coherence tomography (OCT) imaging have greatly improved the characterisation of IRDs. Using wide-field FAF, Oishi *et al*^36^ demonstrated an association between abnormal AF and the severity of functional impairment in COD/CORD, also correlating the extent of reduced autofluorescence with symptom duration.^37^ These findings are substantiated by their correlation with ERG abnormalities.^38^ On OCT, an absent interdigitation zone is an early occurrence in COD and CORD, which is a band representing the interaction between apical processes of the RPE and the photoreceptor OS. Progressive disruption and loss of the ellipsoid zone (EZ) is another notable finding, which corresponds to the ellipsoid portion of the photoreceptor inner segment.^39 40^ Reduction in EZ band reflectance may also be seen in some CODs and CORDs.^41 42^ In advanced disease, outer retinal atrophy including the RPE is observed.

Exploration of phenotypic diversity in COD and CORD has been transformed over the last decade with the application of adaptive optics (AO) in retinal imaging.^42 43^ This technique has enabled imaging of the human retina at a cellular resolution by real-time measurement and correction of individual optical aberrations. Usually integrated with scanning light ophthalmoscopy (SLO), AO imaging has been used to characterise and quantify the central macular photoreceptor mosaic in multiple COD/CORD cohorts. In *GUCY2D*-associated AD-CORD,^44^ for example, residual cone inner segments were visualised at the fovea which were undetected by other imaging modalities. Longitudinal AO imaging thus offers utility in measuring the rate of cone cell loss in progressive disease with precision.^45^

## Specific examples of COD and CORD

### Autosomal dominant *GUCA1A*-associated COD/CORD

#### Molecular genetics

*GUCA1A* is a four-exon gene encoding GCAP1, which is required for RetGC activation and cGMP regeneration.^46^ Since it requires regulation in a Ca^2+^-dependent manner, GCAP1 contains three Ca^2+^-binding EF-hand motifs, structural alterations to which occur in most disease-causing *GUCA1A* sequence variants.^47^ These include the gain-in-function variants p.(Tyr99Cys),^16^ p.(Glu155Gly)^48^ and p.(Asp100Gly).^49^ These result in persistent stimulation of RetGC, excess cGMP levels in the dark and photoreceptor apoptosis secondary to Ca^2+^ dysregulation.^50 51^

**Figure 3:**
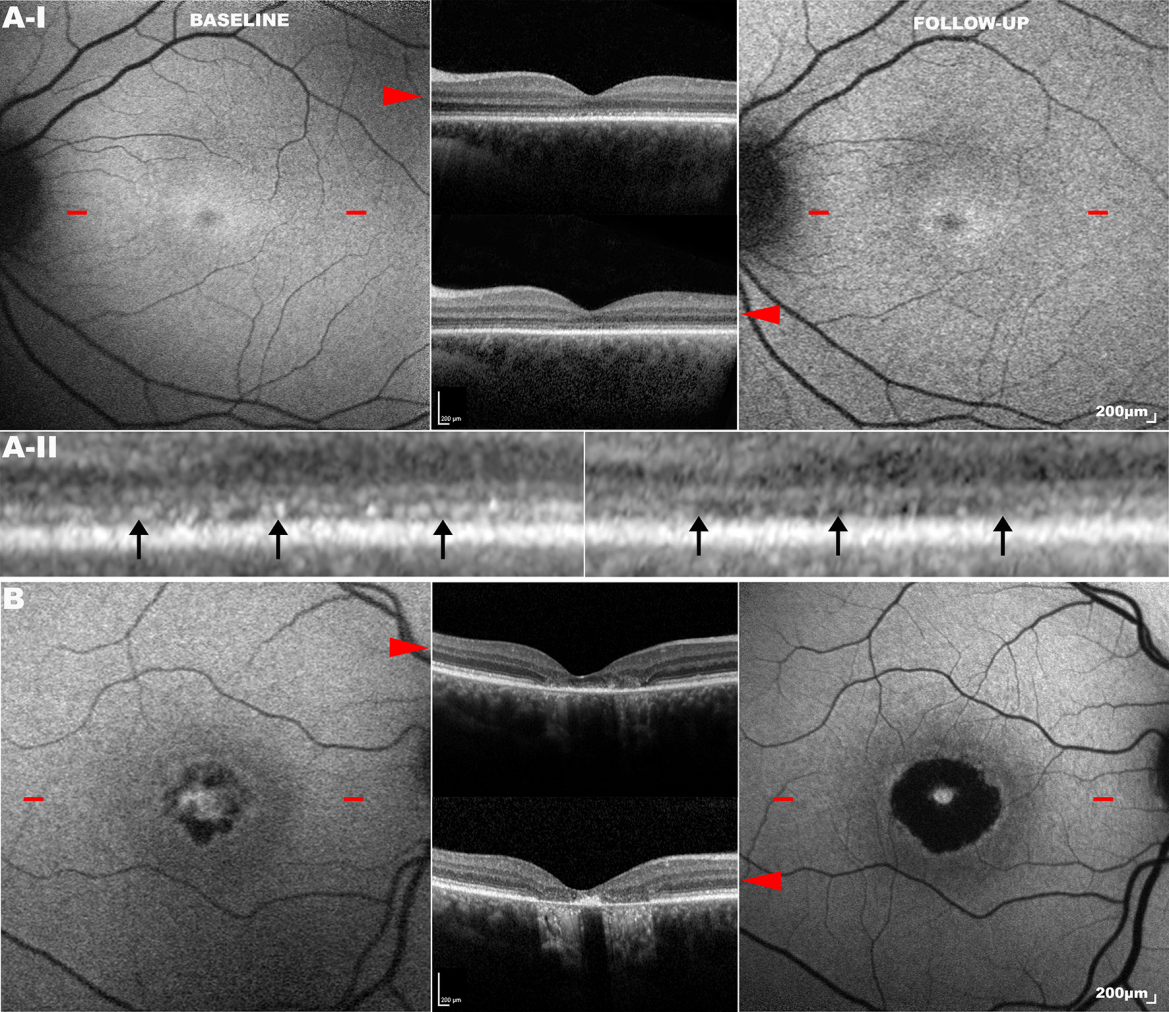
Longitudinal analysis of phenotypically heterogeneous *GUCA1A*-associated retinopathy (p.(Tyr99Cys) substitution). Fundus autofluorescence (FAF) and optical coherence tomography (OCT) imaging in unrelated subjects (A and B) harbouring the p.(Tyr99Cys) (Y99C) variant in *GUCA1A*. The *left column* shows FAF at baseline, and the *right column* that at the same location on follow-up. *Red arrowheads* point to the transfoveal OCT line scan at the location denoted by *red dashes* on FAF. Subject (A): presented with cone dystrophy (A-I baseline) which progressed over a follow-up period of 8 years (A-I follow-up). High magnification (×5) of the same location in the foveal centre (A–II) at baseline and follow-up (*left* and *right*, respectively) shows a greater degree of disruption to the ellipsoid zone (EZ) in the latter (*black arrows*). Subject (B): presented with isolated macular dystrophy which progressed over a 4-year follow-up period. FAF and OCT scans are in the same scale; scale bar=200 µm.

The phenotypic variability in patients harbouring identical *GUCA1A* mutations is noteworthy. In an intrafamilial example, Michaelides *et al*^15^ demonstrated the p.(Tyr99Cys) missense variant resulting in three different dominantly inherited phenotypes in a non-consanguineous British family: COD, CORD and isolated macular dystrophy (figure 3). Similarly, another study identified a p.(Arg120Leu) substitution as the cause of clinically heterogeneous macular dystrophy in a Chinese family, with whole exome sequencing (WES) excluding mutations in other IRD genes.^52^

#### Clinical assessment

Symptomatic onset usually occurs between the second and third decade with reduced central vision, photophobia and generalised dyschromatopsia. ERG studies characteristically show reduced cone single-flash and flicker amplitudes with a normal implicit time (an unusual finding in generalised retinal disease). Rod function typically remains normal, although dysfunction may occur over time in COD or be reduced at presentation in CORD. The relative preservation of rod responses in most *GUCA1A*-associated progressive retinal dystrophies is attributed to greater GCAP1 expression in cones.^53^

#### Retinal imaging

Funduscopy findings are varied, ranging from mild RPE disturbance to extensive macular atrophy. FAF is useful in investigating macular abnormalities, although both areas of hypoautofluorescence and hyperautofluorescence have correlated with retinal atrophy; an increased signal at the fovea may be seen in early disease.^5 15 52^ AOSLO has identified cellular variability between two related patients harbouring a single 428delTinsACAC insertion/deletion variant.^54^ Despite similar clinical findings, they significantly differed in the degree of photoreceptor mosaic disruption, suggesting that other genetic (or environmental) factors influence the effect of the primary disease-causing variant.

### Autosomal dominant *PRPH2*-associated CORD

#### Molecular genetics

*PRPH2* is a five-exon gene encoding peripherin-2, a cell surface glycoprotein in the OS with an essential role in disc morphogenesis.^55^ Interactions of peripherin-2 with ROM1 and glutamic acid-rich domains of CNG channels support its function in disc stabilisation and maintenance of rim curvature.^21^ CORD-associated variants in *PRPH2* can be attributed to the region encoding the second intradiscal loop between its four transmembrane components. This contains cysteine residues essential for intraprotein folding and interprotein interactions.^56^ Identified missense variants in this region include p.(Asn244His),^57^ p.(Val200Glu)^58^ and p.(Arg172Trp).^59^

**Figure 4:**
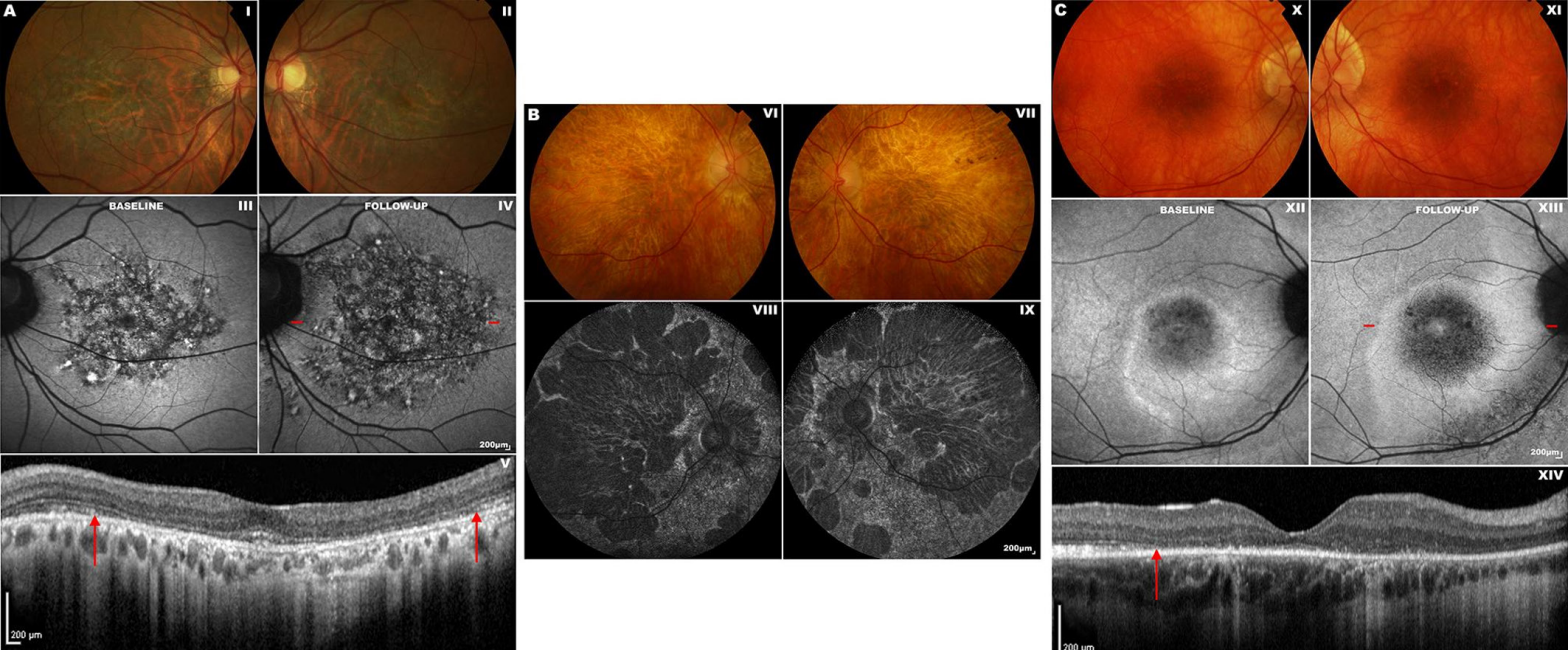
Fundus photography, fundus autofluorescence (FAF) and optical coherence tomography (OCT) imaging in unrelated patients with *PRPH2*-associated and *RPGR*-associated cone-rod dystrophy (CORD). Subjects (A) and (B) possess the p.(Arg172Trp) (R172W) variant in *PRPH2*. Subject (C) possesses the c.2847_2848delinsCT, p.(E950*) variant in *RPGR*. Subject (A): fundus photographs of both eyes (I–II) show bilateral bull’s eye maculopathy-like retinal pigment epithelial (RPE) changes. FAF imaging of the left eye at baseline (III) and on 11-year follow-up (IV) displays a florid speckled appearance with areas of increased and decreased macular autofluorescence. The area of affected retina is substantially increased in (IV), with *red dashes* denoting the location of the OCT scan (V). The inner segment ellipsoid photoreceptor-derived layer (the ellipsoid zone, EZ) between the *red arrows* in (V) is absent. Subject (B): fundus photographs of both eyes (VI–VII) showing marked bilateral macular atrophy, peripheral areas of RPE atrophy and pigmentation. Corresponding FAF images are shown in (VIII–IX). This patient has severe CORD with an acuity of counting fingers bilaterally and constricted peripheral visual fields. Subject (C): fundus photographs of both eyes (X–XI) showing bilateral macular atrophy, which corresponds to the areas of decreased macular autofluorescence. FAF imaging of the right eye at baseline (XII) and on 7-year follow-up (XIII) shows an increase in the hypoautofluorescent area and surrounding hyperautofluorescent ring.^88^ The *red dashes* in (XIII) denote the location of the OCT scan (XIV), in which the temporal border of the photoreceptor layer is marked with a *red arrow*. At its nasal aspect, the integrity of the photoreceptor layer is disrupted.

Families harbouring the p.(Asn244His) or p.(Val200Glu) variant present with early central RPE atrophy that advances peripherally on disease progression, with little intrafamilial variability.^57 58^ In contrast, clinical phenotypes associated with p.(Arg172Trp) can vary substantially, ranging from non-penetrance to severe CORD (figure 4A,B).^59^ In addition to CORD, the p.(Arg172Trp) substitution is associated with other phenotypes, including RP, macular dystrophy and central areolar choroidal dystrophy.^59–62^

#### Clinical assessment

*PRPH2*-associated CORD usually presents in the second to third decade with reduced central vision, photophobia and nyctalopia. Certain genotype–phenotype correlations have been observed, including p.(Arg172Trp)-*PRPH2* retinopathy being associated with faster loss of visual acuity than the p.(Arg172Gln) variant.^62^

#### Retinal imaging

Patients vary considerably in their funduscopic appearance, ranging from a bull’s eye maculopathy (figure 4A) to macular atrophy (figure 4B). However, FAF in *PRPH2*-associated disease, including the p.(Arg172Trp) variant, demonstrates a characteristic speckled macular appearance in most patients (figure 4A, III–IV).^59^ AOSLO imaging in p.(Arg172Trp)-associated CORD revealed increased cone spacing throughout the macula with corresponding loss of outer retinal structures on OCT.^63^ However, intrafamilial analysis of p.(Arg172Gln)-associated disease has shown marked variability, similar to that seen in *GUCA1A*-associated CORD; one family member had a completely normal photoreceptor mosaic, whereas two others had variable parafoveal cone loss.^64^

### Autosomal recessive *ABCA4*-associated COD/CORD

#### Molecular genetics

*ABCA4* is a 50-exon gene that encodes a retina-specific ATP-dependent cassette transporter located in the curved rim of the OS disc membrane.^65^ This protein has an essential role in the removal of *N*-retinylidene-phosphatidylethanolamine (PE) from the luminal to cytoplasmic aspect of the OS disc membrane, which is produced from the reaction of PE with excess chromophores.^9^ If not exported and dissociated, *N*-retinylidene-PE can accumulate in the OS to form the toxic fluorophore, *N*-retinylidene-*N*-retinylethanolamine (A2E), a component of lipofuscin.

*ABCA4* is one of the most common genetic causes of IRD and is associated with vast phenotypic variability. Over 1000 disease-causing variants in *ABCA4* have been identified to date, with the resulting phenotype varying between COD, CORD and Stargardt disease (STGD).^66^ In general, biallelic null variants are more commonly associated with severe and earlier onset CORD and childhood-onset STGD,^67 68^ whereas biallelic missense variants are associated with milder disease such as later onset and foveal-sparing forms of STGD.^69 70^ Functional outcome is dependent on both the variant itself and interaction with other *ABCA4* variants (and other genetic modifiers). For example, p.(Gly1961Glu) in the homozygous state typically results in a milder phenotype,^71^ but can be associated with more severe disease when combined with other variants.^72^

#### Clinical assessment

Symptomatic onset usually occurs in childhood with a central scotoma and rapidly progressing macular atrophy.^68^ The majority of patients have rod involvement at presentation (CORD), which is associated with a worse prognosis.^73^

#### Retinal imaging

**Figure 5:**
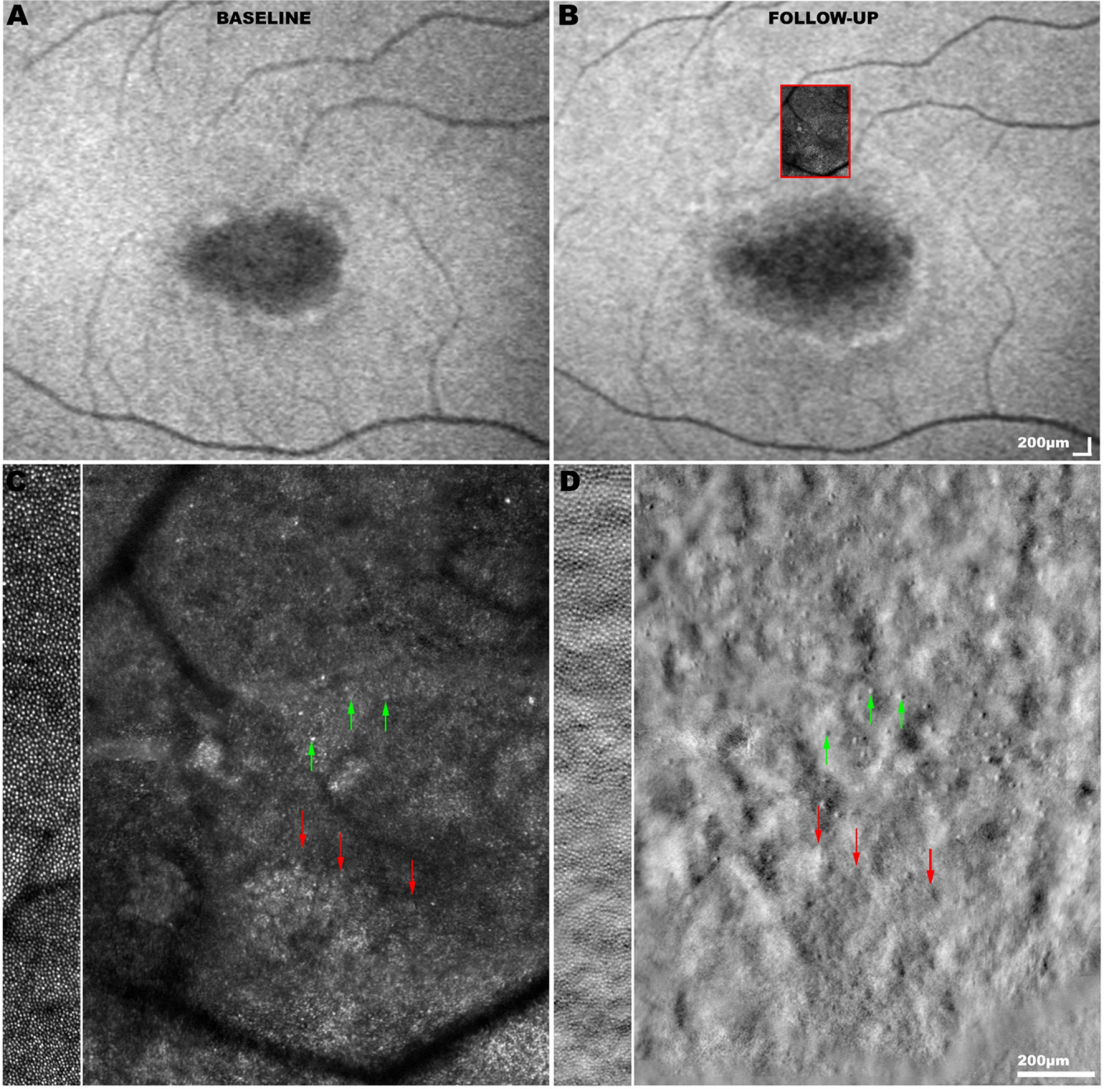
Fundus autofluorescence (FAF) and adaptive optics (AO) imaging in *ABCA4*-associated cone-rod dystrophy (CORD). (A) FAF image at baseline showing a central region of hypoautofluorescence surrounded by increased signal, and (B) aligned FAF image on 2-year follow-up demonstrating disease progression with an increased area of hypoautofluorescence. The *red square* signifies the superimposed adaptive optics scanning light ophthalmoscopy (AOSLO) montage acquired from that region on follow-up. (C) Confocal AOSLO image (photoreceptor outer segments) and (D) split-detection AOSLO image (photoreceptor inner segments) over the transition zone between less affected and more affected retina. For comparison, the side bars on the *left* show AOSLO images of an unaffected control at similar retinal eccentricity. Cone photoreceptors (*green arrows*) can be more reliably identified using split-detection imaging due to the poor wave guiding ability of outer segments in the confocal modality. The border of the transition zone (*red arrows*) in (C) corresponds to the presence of remnant cone inner segments in (D). The photoreceptor mosaic in CORD is disorganised, with altered regularity and reflectance compared to that of an unaffected eye. The number of cones is decreased in areas that appear healthy on FAF, demonstrating a disconnect between imaging modalities and supporting the utility of multimodal imaging. AOSLO images were acquired using a custom-built AOSLO housed at University College London (UCL) / Moorfields Eye Hospital (MEH); scale bar=200 µm.

Funduscopy may initially reveal a normal fundus or mild retinal abnormalities (such as loss of foveal reflex), as peripheral degenerative changes occur in later disease.^74^ Diagnosis can therefore be delayed unless FAF or OCT imaging is undertaken.^68^ FAF findings include a bull’s eye maculopathy-like appearance with yellow-white retinal flecks and increasing macular atrophy over time.^73 75^ OCT reveals loss of outer retinal architecture at the central macula.^76^ Longitudinal increase in abnormal AF regions correlates with both visual functional decline and abnormal cone spacing on AOSLO.^77^ However, cone mosaic abnormalities are known to precede abnormal psychophysical testing and FAF (figure 5).^78^

### X linked *RPGR*-associated COD/CORD

#### Molecular genetics

*RPGR* is a 19-exon gene that gives rise to two alternatively spliced retinal isoforms, encoded by exons 1–19 and 1–15 (+ part of intron 15), respectively.^79^ The latter isoform, also known as exon open reading frame 15 (ORF15), is the most highly expressed retinal variant and a mutational hot spot that accounts for most XL COD and CORD cases.^5 80^ The function of the C-terminal ORF15 sequence requires further elucidation, but is implicated in intraflagellar protein transport in view of its localisation at the photoreceptor connecting cilium.^24^

Most disease-causing variants in *RPGR* result in RP,^81^ but those leading to COD/CORD are preferentially sequestered at the 3’ end of the ORF15 region.^82^ In keeping with the majority of IRD, identical intrafamilial sequence variants in *RPGR* may lead to distinctly different phenotypes.^83^ This is exemplified by a Chinese family harbouring a 2403_04delAG deletion that resulted in both XL-RP and XL-CORD in affected men, reaffirming the importance of disease-modifying factors.^84^

#### Clinical assessment

*RPGR*-associated CORD is characterised by central visual loss, mild photophobia and myopia, and presents in the second to fourth decade in affected men.^85^ A longitudinal study reported significantly higher levels of legal blindness among *RPGR*-associated COD/CORD compared with *RPGR*-associated RP by the age of 40 years; high myopia was predictive of faster visual decline in this study.^86^

#### Retinal imaging

FAF imaging often reveals parafoveal rings of increased signal in *RPGR*-associated COD/CORD. Unlike RP, these increase in size with disease progression (figure 4C, XII–XIII) and are inversely related to ERG amplitude.^87 88^ They are, therefore, a potential endpoint in clinical trials.

## Management of COD and CORD

At present, there are no proven treatments for COD/CORD that halt progression or restore lost vision. Current management consists of symptomatic alleviation, including refractive correction, use of tinted spectacles/contact lenses for photophobia and low vision aids. An accurate diagnosis using molecular genetics is an important step to facilitate genetic counselling, advice on prognosis and participation in anticipated clinical trials.^89^

Patients with specific forms of COD/CORD can be advised to adopt strategies to try to slow degeneration, based on a knowledge of gene function or investigation of animal models. In *GUCA1A*-associated retinopathy, sleeping with the lights on is advocated by some clinicians for preventing accumulation of cGMP, which otherwise occurs at night and causes photoreceptor damage. In contrast, light avoidance using tinted spectacles may confer benefit in *ABCA4*-associated retinopathy by inhibiting A2E production,^90^ which produces DNA-damaging epoxides.^91^ Vitamin A should also be avoided in *ABCA4*-associated retinopathy as it may enhance A2E production and, therefore, disease progression.^92^

Recent evidence has demonstrated the potential of gene therapy for long-term improvement in COD/CORD visual outcomes. Animal models of disease (murine and canine) in *GUCA1A*,^93^ *PRPH2*,^94^ *ABCA4* 95 and *RPGR* 96 have shown significant increase in photoreceptor survival following gene-based therapies. Gene therapy encompasses multiple techniques, including gene replacement, gene editing and gene silencing, with treatment choice dependent on whether the associated sequence variant(s) leads to a loss or gain in function.^97^ Human treatment trials of gene replacement therapy are already under way for retinal disease associated with mutations in *ABCA4* (ClinicalTrials.gov identifier: NCT01367444) and *RPGR* (NCT03252847, NCT03116113 and NCT03316560), with results keenly awaited.

## Limitations of genetic testing

While therapeutic options are on the horizon for COD/CORD secondary to identified disease-causing genes, the outlook for patients without a molecular diagnosis is more limited. NGS-based approaches, typically using WES, have revolutionised genomic analysis, but not all pathogenic mutations can be detected.^98^ Complex changes, such as inversions, translocations and trinucleotide repeat expansions, are mostly undetected with WES, and variants in deep intronic or regulatory regions are not sequenced. Whole genome sequencing (WGS) offers a comprehensive alternative. However, there still remain a proportion of patients who are unsolved despite WGS, due to more complex genetic causes that remain challenging to identify and prove definitively. These include rearrangements and variants in distant promoter/enhancer regions. Over time, improvements in technology and understanding will gradually reduce the number of patients without a genetic diagnosis.^99^

## Concluding remarks and future prospects

Advances in molecular genetic techniques, particularly NGS, have greatly simplified molecular diagnosis. It is hoped that the majority of patients will be able to have a precise molecular diagnosis in the future as the remaining causative genes and sequence variants in CODs and CORDs are identified. Similarly, advances in visual function testing and retinal imaging have improved knowledge of the relationship between genotype and phenotype, which is key to identifying treatment effects in clinical trials of novel therapies. The remaining challenge is to develop novel therapies that will slow degeneration or improve function, and it is encouraging that gene-based approaches to therapy are increasingly in clinical trial with the first Food and Drug Administration-approved gene therapy for LCA-*RPE65* now available.
