# Supplementary material for: Progressive cone and cone-rod dystrophies: clinical features, molecular genetics and prospects for therapy
Source: Br J Ophthalmol. 2019 Jan 24;103(5):711–20. doi: 10.1136/bjophthalmol-2018-313278 (PMC6709772; doi:10.1136/bjophthalmol-2018-313278)
Supplement: Supplementary data [file bjophthalmol-2018-313278supp001.pdf]

Supplementary Table 1: Occurrence and frequency of mutations in autosomal dominant (AD) cone and cone-rod dystrophy

STUDY	*AIPL1*	*CRX*	*GUCA1A*	*GUCY2D*	*PITPNM3*	*PROM1*	*PRPH2*	*RAX2*	*RIMS1*	*UNC119*
**(Sohocki et al., 2000b)**	1/15									
**(Boulanger-scemama et al., 2015)**	2/96	4/96	1/96	5/96		9/96	2/96			
**(Freund et al., 1997)**		2/30								
**(Swain et al., 1997)**		4								
**(Sohocki et al., 1998)**		3/30								
**(Jacobson et al., 1998)**		1								
**(Sankila et al., 2000)**		1								
**(Sohocki et al., 2000a)**		4/67								
**(Rivolta et al., 2001)**		0/88								
**(Lines et al., 2002)**		1								
**(Itabashi et al., 2004)**		1								
**(Henderson et al., 2007)**		1/29								
**(Paunescu et al., 2007)**		1								
**(Kitiratschky et al., 2008b)**		1								
**(Kitiratschky et al., 2011)**		1/7	3/22	3/7			2/22			
**(Kohl et al., 2012)**		2/52	4/52	12/52		1/52	6/52			
**(Huang et al., 2012)**		3/130								
**(Lu et al., 2015)**		1								
**(Huang et al., 2016)**		3/163	2/163	5/163						1/163
**(Oishi et al., 2016)**		2/43		1/43		2/43	1/43			
**(Birtel et al., 2018)**		4/251	3/251	6/251		6/251	29/251			
**(Payne et al., 1998)**			1							
**(Downes et al., 2001a)**			3							
**(Wilkie et al., 2001)**			1							
**(Nishiguchi et al., 2004)**			1/119							
**(Jiang et al., 2005)**			1							
**(Michaelides et al., 2005)**			1							
**(Sokal et al., 2005)**			1							
**(Jiang et al., 2008)**			1							
**(Kitiratschky et al., 2009)**			4/24							
**(Kamenarova et al., 2013)**			2							
**(Huang et al., 2013a)**			1							
**(Nong et al., 2014)**			1							
**(Manes et al., 2017)**			2							
**(Marino et al., 2018)**			1							
**(Kelsell et al., 1998)**				4						
**(Gregory-evans et al., 2000)**				1						
**(Weigell-weber et al., 2000)**				1						

STUDY	*AIPL1*	*CRX*	*GUCA1A*	*GUCY2D*	*PITPNM3*	*PROM1*	*PRPH2*	*RAX2*	*RIMS1*	*UNC119*
**(Van ghelue et al., 2001)**				1						
**(Downes et al., 2001b)**				4						
**(Payne et al., 2001)**				3/40						
**(Udar et al., 2003)**				2						
**(Ito et al., 2004a)**				3/38						
**(Ito et al., 2004b)**				2						
**(Yoshida et al., 2006)**				1						
**(Smith et al., 2006)**				1						
**(Kitiratschky et al., 2008c)**				11/27						
**(Small et al., 2008)**				1						
**(Garcia-hoyos et al., 2011)**				2/22						
**(Xiao et al., 2011)**				1						
**(Xu et al., 2013)**				1						
**(Huang et al., 2013b)**				2/130			1/130			1/130
**(Zhao et al., 2013)**				1						
**(Alapati et al., 2014)**				6/54						
**(Lazar et al., 2015)**				2/6						
**(Jiang et al., 2015)**				7/15						
**(Köhn et al., 2007)**					2					
**(Köhn et al., 2010)**					5/163					
**(Yang et al., 2008)**						3				
**(Michaelides et al., 2010)**						5				
**(Eidinger et al., 2015)**						1				
**(Mayer et al., 2016)**						1				
**(Wawrocka et al., 2018)**						1/18	1/18			
**(Nakazawa et al., 1996a)**							4			
**(Nakazawa et al., 1996b)**							1			
**(Renner et al., 2009)**							3			
**(Yang et al., 2015)**								1		
**(Johnson et al., 2003)**									1	
**(Kobayashi et al., 2000)**										1/20
**OVERALL MUTATION FREQUENCY**	3/111 (1.2%)	40/997 (15.6%)	34/743 (13.2%)	89/965 (34.6%)	7/165 (2.7%)	29/470 (11.3%)	50/620 (19.5%)	1/1 (0.4%)	1/1 (0.4%)	3/313 (1.2%)

Note: Denominators represent the total number of unrelated probands with a clinical diagnosis of progressive cone dystrophy (COD) or cone-rod dystrophy (CORD) in each patient cohort. Numerators represent the number of patients with disease secondary to pathogenic variant/s in each respective gene. Non-fractional integers represent the number of unique pathogenic variants identified in a gene from pedigree analysis.

Percentages of overall mutation frequency represent the proportion of cases each gene is responsible for from the total number of identified genetic variants in a single mode of inheritance.

Supplementary Table 2: Occurrence and frequency of mutations in autosomal recessive (AR) cone and cone-rod dystrophy

STUDY	*ABCA4*	*ADAM9*	*C21ORF2*	*C8ORF37*	*CACNA2D4*	*CDHR1*	*CEP78*	*CERKL*	*CNGA3*	*CNGB3*	*KCNV2*	*PDE6C*	*PDE6H*	*POC1B*	*RAB28*	*RPGRIP1*	*SEMA4A*	*TTLL5*
**(Cremers et al., 1998)**	1																	
**(Maugeri et al., 2000)**	13/20																	
**(Papaioannou et al., 2000)**	4/8																	
**(Birch et al., 2001)**	11/37																	
**(Briggs et al., 2001)**	5/8																	
**(Paloma et al., 2001)**	2																	
**(Ducroq et al., 2002)**	13/55																	
**(Paloma et al., 2002)**	1																	
**(Fishman et al., 2003)**	16/30																	
**(Klevering et al., 2004a)**	1																	
**(Klevering et al., 2004b)**	18/54																	
**(Simonelli et al., 2004)**	1																	
**(Klevering et al., 2005)**	2																	
**(Ducroq et al., 2006)**	1																	
**(Stenirri et al., 2006)**	1																	
**(Singh et al., 2006)**	2/14																	
**(Michaelides et al., 2007)**	4																	
**(Yzer et al., 2007)**	1																	
**(Kitiratschky et al., 2008a)**	20/64																	
**(Ernest et al., 2009)**	3																	
**(Xi et al., 2009)**	1																	
**(Aguirre-lamban et al., 2009)**	1/5																	
**(Littink et al., 2010)**	33/123							2/123			1/123							
**(Thiadens et al., 2012)**	25/155									3/90	4/90							
**(Huang et al., 2013c)**	1/47									3/47		2/47				1/47		
**(Bocquet et al., 2013)**	16/34																	
**(Riveiro-alvarez et al., 2013)**	26/86																	
**(Alapati et al., 2014)**	15/54																	
**(Boulanger-scemama et al., 2015)**	23/96		2/96			1/96		1/96				4/96				1/96	2/96	
**(Huang et al., 2016)**	6/163		1/163						53/163	3/163	1/163	3/163				2/163		
**(Oishi et al., 2016)**	4/43					1/43												
**(Birtel et al., 2018)**	94/251					6/251		2/251						1/251			1/251	
**(Parry et al., 2009)**		4																
**(El-haig et al., 2014)**		1																
**(Hull et al., 2014)**		1																

STUDY	*ABCA4*	*ADAM9*	*C21ORF2*	*C8ORF37*	*CACNA2D4*	*CDHR1*	*CEP78*	*CERKL*	*CNGA3*	*CNGB3*	*KCNV2*	*PDE6C*	*PDE6H*	*POC1B*	*RAB28*	*RPGRIP1*	*SEMA4A*	*TTLL5*
**(Khan et al., 2015)**			3															
**(Suga et al., 2016)**			2															
**(Estrada-cuzcano et al., 2012)**				4														
**(Lazar et al., 2015)**				1/6		1/6												
**(Rahner et al., 2016)**				1														
**(Katagiri et al., 2016)**				2														
**(Wycisk et al., 2006)**					1/34													
**(Ba-abbad et al., 2016)**					2													
**(Ostergaard et al., 2010)**						1												
**(Henderson et al., 2010)**						2												
**(Cohen et al., 2012)**						1												
**(Fu et al., 2018)**						1												
**(Nikopoulos et al., 2016)**							2											
**(Namburi et al., 2016)**							5											
**(Fu et al., 2017)**							2											
**(Auslender et al., 2007)**								7/21										
**(Ali et al., 2008)**								1										
**(Aleman et al., 2009)**								3										
**(Wissinger et al., 2001)**									3/48									
**(Shaikh et al., 2015)**									1									
**(Michaelides et al., 2004)**										4								
**(Thiadens et al., 2010)**										3/60								
**(Wu et al., 2006)**											10/11							
**(Thiagalingam et al., 2007)**											8							
**(Salah et al., 2008)**											3							
**(Wissinger et al., 2008)**											11							
**(Wissinger et al., 2011)**											4/154							
**(Fujinami et al., 2013)**											2							
**(Thiadens et al., 2009)**												1/104						
**(Piri et al., 2005)**													1					
**(Durlu et al., 2014)**														1				
**(Roosing et al., 2014)**														2				
**(Jin et al., 2018)**														1				
**(Roosing et al., 2013)**															2/617			
**(Riveiro-álvarez et al., 2015)**															2/109			
**(Hameed et al., 2003)**																2		
**(Abid et al., 2006)**																	2/25	
**(Sergouniotis et al., 2014)**																		4/83

<u>STUDY</u>	***ABCA4***	***ADAM9***	***C21ORF2***	***C8ORF37***	***CACNA2D4***	***CDHR1***	***CEP78***	***CERKL***	***CNGA3***	***CNGB3***	***KCNV2***	***PDE6C***	***PDE6H***	***POC1B***	***RAB28***	***RPGRIP1***	***SEMA4A***	***TTLL5***
**(Bedoni et al., 2016)**																		6
**OVERALL MUTATION FREQUENCY**	365/ 1366 (62.2%)	6/6 (1.0%)	8/264 (1.4%)	8/13 (1.4%)	3/36 (0.5%)	14/401 (2.4%)	9/9 (1.5%)	16/495 (2.7%)	57/212 (9.7%)	16/364 (2.7%)	44/565 (7.5%)	10/410 (1.7%)	1/1 (0.2%)	5/255 (0.9%)	4/726 (0.7%)	6/308 (1.0%)	5/372 (0.9%)	10/89 (1.7%)

Supplementary Table 3: Occurrence and frequency of mutations in X-linked (XL) cone and cone-rod dystrophy

STUDY	*CACNA1F*	*OPN1LW / OPN1MW*	*RPGR*
**(Jalkanen et al., 2006)**	1		
**(Huang et al., 2013c)**	1/47		2/47
**(Hauke et al., 2013)**	1		
**(Huang et al., 2016)**	4/163		
**(Gardner et al., 2010)**		2	
**(Gardner et al., 2012)**		1	
**(Demirci et al., 2002)**			3
**(Yang et al., 2002)**			2
**(Ebenezer et al., 2005)**			2/6
**(Ruddle et al., 2009)**			3
**(Thiadens et al., 2012)**			7/8
**(Bocquet et al., 2013)**			2/10
**(Birtel et al., 2018)**			1/251
**(Wawrocka et al., 2018)**			5/18
**OVERALL MUTATION FREQUENCY**	7/212 (18.9%)	3/3 (8.1%)	27/348 (73.0%)

References

Abid, A., Ismail, M., Mehdi, S. Q., et al. 2006. Identification of Novel Mutations in the SEMA4A Gene Associated with Retinal Degenerative Diseases. *Journal of Medical Genetics,* 43**,** 378-381.

Aguirre-Lamban, J., Riveiro-Alvarez, R., Maia-Lopes, S., et al. 2009. Molecular Analysis of the ABCA4 Gene for Reliable Detection of Allelic Variations in Spanish Patients: Identification of 21 Novel Variants. *The British Journal of Ophthalmology,* 93**,** 614-621.

Alapati, A., Goetz, K., Suk, J., et al. 2014. Molecular Diagnostic Testing By EyeGENE: Analysis of Patients with Hereditary Retinal Dystrophy Phenotypes Involving Central Vision Loss. *Investigative Ophthalmology & Visual Science,* 55**,** 5510-5521.

Aleman, T. S., Soumittra, N., Cideciyan, A. V., et al. 2009. CERKL Mutations Cause an Autosomal Recessive Cone-Rod Dystrophy with Inner Retinopathy. *Investigative Ophthalmology & Visual Science,* 50**,** 5944-5954.

Ali, M., Ramprasad, V. L., Soumittra, N., et al. 2008. A Missense Mutation in the Nuclear Localization Signal Sequence of CERKL (p.R106S) Causes Autosomal Recessive Retinal Degeneration. *Molecular Vision,* 14**,** 1960-1964.

Auslender, N., Sharon, D., Abbasi, A. H., et al. 2007. A Common Founder Mutation of CERKL Underlies Autosomal Recessive Retinal Degeneration with Early Macular Involvement among Yemenite Jews. *Investigative Ophthalmology & Visual Science,* 48**,** 5431-5438.

Ba-Abbad, R., Arno, G., Carss, K., et al. 2016. Mutations in CACNA2D4 Cause Distinctive Retinal Dysfunction in Humans. *Ophthalmology,* 123**,** 668-671.e2.

Bedoni, N., Haer-Wigman, L., Vaclavik, V., et al. 2016. Mutations in the Polyglutamylase Gene TTLL5, Expressed in Photoreceptor Cells and Spermatozoa, are Associated with Cone-Rod Degeneration and Reduced Male Fertility. *Human Molecular Genetics,* 25**,** 4546-4555.

Birch, D. G., Peters, A. Y., Locke, K. L., et al. 2001. Visual Function in Patients with Cone–Rod Dystrophy (CRD) Associated with Mutations in the ABCA4 (ABCR) Gene. *Experimental Eye Research,* 73**,** 877-886.

Birtel, J., Eisenberger, T., Gliem, M., et al. 2018. Clinical and Genetic Characteristics of 251 Consecutive Patients with Macular and Cone/Cone-Rod Dystrophy. *Scientific Reports,* 8**,** 4824-4824.

Bocquet, B., Lacroux, A., Surget, M.-O., et al. 2013. Relative Frequencies of Inherited Retinal Dystrophies and Optic Neuropathies in Southern France: Assessment of 21-year Data Management. *Ophthalmic Epidemiology,* 20**,** 13-25.

Boulanger-Scemama, E., El Shamieh, S., Démontant, V., et al. 2015. Next-Generation Sequencing Applied to a Large French Cone and Cone-Rod Dystrophy Cohort: Mutation Spectrum and New Genotype-Phenotype Correlation. *Orphanet Journal of Rare Diseases,* 10**,** 85-85.

Briggs, C. E., Rucinski, D., Rosenfeld, P. J., et al. 2001. Mutations in ABCR (ABCA4) in Patients with Stargardt Macular Degeneration or Cone-Rod Degeneration. *Investigative Ophthalmology & Visual Science,* 42**,** 2229-2236.

Cohen, B., Chervinsky, E., Jabaly-Habib, H., et al. 2012. A Novel Splice Site Mutation of CDHR1 in a Consanguineous Israeli Christian Arab Family Segregating Autosomal Recessive Cone-Rod Dystrophy. *Molecular Vision,* 18**,** 2915-2921.

Cremers, F. P. M., Van De Pol, D. J. R., Van Driel, M., et al. 1998. Autosomal Recessive Retinitis Pigmentosa and Cone-Rod Dystrophy Caused by Splice Site Mutations in the Stargardt's Disease Gene ABCR. *Human Molecular Genetics,* 7**,** 355-362.

Demirci, F. Y. K., Rigatti, B. W., Wen, G., et al. 2002. X-Linked Cone-Rod Dystrophy (Locus COD1): Identification of Mutations in RPGR Exon ORF15. *American Journal of Human Genetics,* 70**,** 1049-1053.

Downes, S. M., Holder, G. E., Fitzke, F. W., et al. 2001a. Autosomal dominant cone and cone-rod dystrophy with mutations in the guanylate cyclase activator 1a gene-encoding guanylate cyclase activating protein-1. *Archives of Ophthalmology,* 119**,** 96-105.

Downes, S. M., Payne, A. M., Kelsell, R. E., et al. 2001b. Autosomal dominant cone-rod dystrophy with mutations in the guanylate cyclase 2d gene encoding retinal guanylate cyclase-1. *Archives of Ophthalmology,* 119**,** 1667-1673.

Ducroq, D., Rozet, J.-M., Gerber, S., et al. 2002. The ABCA4 gene in autosomal recessive cone-rod dystrophies. *American Journal of Human Genetics,* 71**,** 1480-1482.

Ducroq, D., Shalev, S., Habib, A., et al. 2006. Three different ABCA4 mutations in the same large family with several consanguineous loops affected with autosomal recessive cone–rod dystrophy. *European Journal Of Human Genetics,* 14**,** 1269.

Durlu, Y. K., Köroğlu, Ç. & Tolun, A. 2014. Novel recessive cone-rod dystrophy caused by POC1B mutation. *JAMA Ophthalmology,* 132**,** 1185-1191.

Ebenezer, N. D., Michaelides, M., Jenkins, S. A., et al. 2005. Identification of novel RPGR ORF15 mutations in X-linked progressive cone-rod dystrophy (XLCORD) families. *Investigative Ophthalmology & Visual Science,* 46**,** 1891-8.

Eidinger, O., Leibu, R., Newman, H., et al. 2015. An intronic deletion in the PROM1 gene leads to autosomal recessive cone-rod dystrophy. *Molecular Vision,* 21**,** 1295-1306.

El-Haig, W. M., Jakobsson, C., Favez, T., et al. 2014. Novel ADAM9 homozygous mutation in a consanguineous Egyptian family with severe cone-rod dystrophy and cataract. *British Journal of Ophthalmology,* 98**,** 1718.

Ernest, P. J. G., Boon, C. J. F., Klevering, B. J., et al. 2009. Outcome of ABCA4 microarray screening in routine clinical practice. *Molecular Vision,* 15**,** 2841-2847.

Estrada-Cuzcano, A., Neveling, K., Kohl, S., et al. 2012. Mutations in C8orf37, encoding a ciliary protein, are associated with autosomal-recessive retinal dystrophies with early macular involvement. *American Journal of Human Genetics,* 90**,** 102-9.

Fishman, G. A., Stone, E. M., Eliason, D. A., et al. 2003. ABCA4 gene sequence variations in patients with autosomal recessive cone-rod dystrophy. *Archives of Ophthalmology,* 121**,** 851-855.

Freund, C. L., Gregory-Evans, C. Y., Furukawa, T., et al. 1997. Cone-Rod Dystrophy Due to Mutations in a Novel Photoreceptor-Specific Homeobox Gene (CRX) Essential for Maintenance of the Photoreceptor. *Cell,* 91**,** 543-553.

Fu, J., Ma, L., Cheng, J., et al. 2018. A novel, homozygous nonsense variant of the CDHR1 gene in a Chinese family causes autosomal recessive retinal dystrophy by NGS-based genetic diagnosis. *Journal of Cellular and Molecular Medicine,* 22**,** 5662-5669.

Fu, Q., Xu, M., Chen, X., et al. 2017. CEP78 is mutated in a distinct type of Usher syndrome. *Journal of Medical Genetics,* 54**,** 190.

Fujinami, K., Tsunoda, K., Nakamura, N., et al. 2013. Molecular characteristics of four Japanese cases with KCNV2 retinopathy: report of novel disease-causing variants. *Molecular Vision,* 19**,** 1580-1590.

Garcia-Hoyos, M., Auz-Alexandre, C. L., Almoguera, B., et al. 2011. Mutation analysis at codon 838 of the Guanylate Cyclase 2D gene in Spanish families with autosomal dominant cone, cone-rod, and macular dystrophies. *Molecular Vision,* 17**,** 1103-1109.

Gardner, J. C., Webb, T. R., Kanuga, N., et al. 2010. X-linked cone dystrophy caused by mutation of the red and green cone opsins. *American Journal of Human Genetics,* 87**,** 26-39.

Gardner, J. C., Webb, T. R., Kanuga, N., et al. 2012. A novel missense mutation in both OPN1LW and OPN1MW cone opsin genes causes X-linked cone dystrophy (XLCOD5). *Adv Exp Med Biol,* 723**,** 595-601.

Gregory-Evans, K., Kelsell, R. E., Gregory-Evans, C. Y., et al. 2000. Autosomal dominant cone-rod retinal dystrophy (CORD6) from heterozygous mutation of GUCY2D, which encodes retinal guanylate cyclase 1. *Ophthalmology,* 107**,** 55-61.

Hameed, A., Abid, A., Aziz, A., et al. 2003. Evidence of RPGRIP1 gene mutations associated with recessive cone-rod dystrophy. *Journal of Medical Genetics,* 40**,** 616-619.

Hauke, J., Schild, A., Neugebauer, A., et al. 2013. A novel large in-frame deletion within the CACNA1F gene associates with a cone-rod dystrophy 3-like phenotype. *PLOS ONE,* 8**,** e76414-e76414.

Henderson, R. H., Li, Z., Abd El Aziz, M. M., et al. 2010. Biallelic mutation of protocadherin-21 (PCDH21) causes retinal degeneration in humans. *Molecular Vision,* 16**,** 46-52.

Henderson, R. H., Waseem, N., Searle, R., et al. 2007. An Assessment of the Apex Microarray Technology in Genotyping Patients with Leber Congenital Amaurosis and Early-Onset Severe Retinal Dystrophy. *Investigative Ophthalmology & Visual Science,* 48**,** 5684-5689.

Huang, L., Li, S., Xiao, X., et al. 2013a. Novel GUCA1A mutation identified in a Chinese family with cone-rod dystrophy. *Neuroscience Letters,* 541**,** 179-183.

Huang, L., Li, S., Xiao, X., et al. 2013b. Screening for variants in 20 genes in 130 unrelated patients with cone-rod dystrophy. *Molecular Medicine Reports,* 7.

Huang, L., Xiao, X., Li, S., et al. 2012. CRX variants in cone–rod dystrophy and mutation overview. *Biochemical and Biophysical Research Communications,* 426**,** 498-503.

Huang, L., Xiao, X., Li, S., et al. 2016. Molecular genetics of cone-rod dystrophy in Chinese patients: New data from 61 probands and mutation overview of 163 probands. *Experimental Eye Research,* 146**,** 252-258.

Huang, L., Zhang, Q., Li, S., et al. 2013c. Exome Sequencing of 47 Chinese Families with Cone-Rod Dystrophy: Mutations in 25 Known Causative Genes. *PLOS ONE,* 8**,** e65546.

Hull, S., Arno, G., Plagnol, V., et al. 2014. Exome sequencing reveals ADAM9 mutations in a child with cone-rod dystrophy. *Acta Ophthalmologica,* 93**,** e392-e393.

Itabashi, T., Wada, Y., Sato, H., et al. 2004. Novel 615delC mutation in the CRX gene in a Japanese family with cone-rod dystrophy. *American Journal of Ophthalmology,* 138**,** 876-877.

Ito, S., Nakamura, M., Nuno, Y., et al. 2004a. Novel Complex GUCY2D Mutation in Japanese Family with Cone-Rod Dystrophy. *Investigative Ophthalmology & Visual Science,* 45**,** 1480-1485.

Ito, S., Nakamura, M., Ohnishi, Y., et al. 2004b. Autosomal Dominant Cone-Rod Dystrophy with R838H and R838C Mutations in the GUCY2D Gene in Japanese Patients. *Japanese Journal of Ophthalmology,* 48**,** 228-235.

Jacobson, S. G., Cideciyan, A. V., Huang, Y., et al. 1998. Retinal degenerations with truncation mutations in the cone-rod homeobox (CRX) gene. *Investigative Ophthalmology & Visual Science,* 39**,** 2417-2426.

Jalkanen, R., Mäntyjärvi, M., Tobias, R., et al. 2006. X linked cone-rod dystrophy, CORDX3, is caused by a mutation in the CACNA1F gene. *Journal of Medical Genetics,* 43**,** 699-704.

Jiang, F., Xu, K., Zhang, X., et al. 2015. GUCY2D mutations in a Chinese cohort with autosomal dominant cone or cone–rod dystrophies. *Documenta Ophthalmologica,* 131**,** 105-114.

Jiang, L., J Katz, B., Yang, Z., et al. 2005. Autosomal dominant cone dystrophy caused by a novel mutation in the GCAP1 gene (GUCA1A). *Molecular Vision,* 11**,** 143-51.

Jiang, L., Wheaton, D., Bereta, G., et al. 2008. A novel GCAP1(N104K) mutation in EF-hand 3 (EF3) linked to autosomal dominant cone dystrophy. *Vision Research,* 48**,** 2425-2432.

Jin, X., Chen, L., Wang, D., et al. 2018. Novel compound heterozygous mutation in the POC1B gene underlie peripheral cone dystrophy in a Chinese family. *Ophthalmic Genetics,* 39**,** 300-306.

Johnson, S., Halford, S., Morris, A. G., et al. 2003. Genomic organisation and alternative splicing of human RIM1, a gene implicated in autosomal dominant cone-rod dystrophy (CORD7). *Genomics,* 81**,** 304-314.

Kamenarova, K., Corton, M., García-Sandoval, B., et al. 2013. Novel GUCA1A mutations suggesting possible mechanisms of pathogenesis in cone, cone-rod, and macular dystrophy patients. *BioMed Research International,* 2013**,** 517570-517570.

Katagiri, S., Hayashi, T., Yoshitake, K., et al. 2016. Novel C8orf37 Mutations in Patients with Early-onset Retinal Dystrophy, Macular Atrophy, Cataracts, and High Myopia. *Ophthalmic Genetics,* 37**,** 68-75.

Kelsell, R. E., Gregory-Evans, K., Payne, A. M., et al. 1998. Mutations in the Retinal Guanylate Cyclase (RETGC-1) Gene in Dominant Cone-Rod Dystrophy. *Human Molecular Genetics,* 7**,** 1179-1184.

Khan, A. O., Eisenberger, T., Nagel-Wolfrum, K., et al. 2015. C21orf2 is mutated in recessive early-onset retinal dystrophy with macular staphyloma and encodes a protein that localises to the photoreceptor primary cilium. *British Journal of Ophthalmology,* 99**,** 1725.

Kitiratschky, V. B. D., Behnen, P., Kellner, U., et al. 2009. Mutations in the GUCA1A gene involved in hereditary cone dystrophies impair calcium-mediated regulation of guanylate cyclase. *Human Mutation,* 30**,** E782-E796.

Kitiratschky, V. B. D., Glöckner, C. J. & Kohl, S. 2011. Mutation Screening of the GUCA1B Gene in Patients with Autosomal Dominant Cone and Cone Rod Dystrophy. *Ophthalmic Genetics,* 32**,** 151-155.

Kitiratschky, V. B. D., Grau, T., Bernd, A., et al. 2008a. ABCA4 gene analysis in patients with autosomal recessive cone and cone rod dystrophies. *European Journal Of Human Genetics,* 16**,** 812-819.

Kitiratschky, V. B. D., Nagy, D., Zabel, T., et al. 2008b. Cone and cone–rod dystrophy segregating in the same pedigree due to the same novel CRX gene mutation. *British Journal of Ophthalmology,* 92**,** 1086.

Kitiratschky, V. B. D., Wilke, R., Renner, A. B., et al. 2008c. Mutation analysis identifies GUCY2D as the major gene responsible for autosomal dominant progressive cone degeneration. *Investigative Ophthalmology & Visual Science,* 49**,** 5015-5023.

Klevering, B. J., Deutman, A. F., Maugeri, A., et al. 2005. The spectrum of retinal phenotypes caused by mutations in the ABCA4 gene. *Graefe's Archive for Clinical and Experimental Ophthalmology,* 243**,** 90-100.

Klevering, B. J., Maugeri, A., Wagner, A., et al. 2004a. Three families displaying the combination of Stargardt's disease with cone-rod dystrophy or retinitis pigmentosa. *Ophthalmology,* 111**,** 546-553.

Klevering, B. J., Yzer, S., Rohrschneider, K., et al. 2004b. Microarray-based mutation analysis of the ABCA4 (ABCR) gene in autosomal recessive cone–rod dystrophy and retinitis pigmentosa. *European Journal Of Human Genetics,* 12**,** 1024.

Kobayashi, A., Higashide, T., Hamasaki, D., et al. 2000. HRG4 (UNC119) Mutation Found in Cone–Rod Dystrophy Causes Retinal Degeneration in a Transgenic Model. *Investigative Ophthalmology & Visual Science,* 41**,** 3268-3277.

Kohl, S., Kitiratschky, V., Papke, M., et al. Genes and Mutations in Autosomal Dominant Cone and Cone-Rod Dystrophy. *In:* LAVAIL, M. M., ASH, J. D., ANDERSON, R. E., HOLLYFIELD, J. G. & GRIMM, C., eds. Retinal Degenerative Diseases, 2012// 2012 Boston, MA. Springer US, 337-343.

Köhn, L., Kadzhaev, K., Burstedt, M. S. I., et al. 2007. Mutation in the PYK2-binding domain of PITPNM3 causes autosomal dominant cone dystrophy (CORD5) in two Swedish families. *European Journal Of Human Genetics,* 15**,** 664.

Köhn, L., Kohl, S., Bowne, S. J., et al. 2010. PITPNM3 is an uncommon cause of cone and cone-rod dystrophies. *Ophthalmic Genetics,* 31**,** 139-140.

Lazar, C. H., Mutsuddi, M., Kimchi, A., et al. 2015. Whole exome sequencing reveals GUCY2D as a major gene associated with cone and cone-rod dystrophy in Israel. *Investigative Ophthalmology & Visual Science,* 56**,** 420-430.

Lines, M. A., Hébert, M., Mctaggart, K. E., et al. 2002. Electrophysiologic and phenotypic features of an autosomal cone-rod dystrophy caused by a novel CRX mutation. *Ophthalmology,* 109**,** 1862-1870.

Littink, K. W., Koenekoop, R. K., Van Den Born, L. I., et al. 2010. Homozygosity mapping in patients with cone-rod dystrophy: novel mutations and clinical characterizations. *Investigative Ophthalmology & Visual Science,* 51**,** 5943-5951.

Lu, Q.-K., Zhao, N., Lv, Y.-S., et al. 2015. A novel CRX mutation by whole-exome sequencing in an autosomal dominant cone-rod dystrophy pedigree. *International Journal of Ophthalmology,* 8**,** 1112-1117.

Manes, G., Mamouni, S., Hérald, E., et al. 2017. Cone dystrophy or macular dystrophy associated with novel autosomal dominant GUCA1A mutations. *Molecular Vision,* 23**,** 198-209.

Marino, V., Dal Cortivo, G., Oppici, E., et al. 2018. A novel p.(Glu111Val) missense mutation in GUCA1A associated with cone-rod dystrophy leads to impaired calcium sensing and perturbed second messenger homeostasis in photoreceptors. *Human Molecular Genetics*, ddy311-ddy311.

Maugeri, A., Klevering, B. J., Rohrschneider, K., et al. 2000. Mutations in the ABCA4 (ABCR) gene are the major cause of autosomal recessive cone-rod dystrophy. *American Journal of Human Genetics,* 67**,** 960-966.

Mayer, A. K., Rohrschneider, K., Strom, T. M., et al. 2016. Homozygosity mapping and whole-genome sequencing reveals a deep intronic PROM1 mutation causing cone-rod dystrophy by pseudoexon activation. *European Journal of Human Genetics,* 24**,** 459-462.

Michaelides, M., Chen, L. L., Brantley, M. A., Jr., et al. 2007. ABCA4 mutations and discordant ABCA4 alleles in patients and siblings with bull's-eye maculopathy. *British Journal of Ophthalmology,* 91**,** 1650-1655.

Michaelides, M., Gaillard, M.-C., Escher, P., et al. 2010. The PROM1 mutation p.R373C causes an autosomal dominant bull's eye maculopathy associated with rod, rod-cone, and macular dystrophy. *Investigative Ophthalmology & Visual Science,* 51**,** 4771-4780.

Michaelides, M., Hunt, D. M. & Moore, A. T. 2004. The cone dysfunction syndromes. *British Journal of Ophthalmology,* 88**,** 291-7.

Michaelides, M., Wilkie, S. E., Jenkins, S., et al. 2005. Mutation in the gene GUCA1A, encoding guanylate cyclase-activating protein 1, causes cone, cone-rod, and macular dystrophy. *Ophthalmology,* 112**,** 1442-7.

Nakazawa, M., Kikawa, E., Chida, Y., et al. 1996a. Autosomal dominant cone-rod dystrophy associated with mutations in codon 244 (asn244his) and codon 184 (tyr184ser) of the peripherin/rds gene. *Archives of Ophthalmology,* 114**,** 72-78.

Nakazawa, M., Naoi, N., Wada, Y., et al. 1996b. Autosomal dominant cone-rod dystrophy associated with a Val200GLu mutation of the peripherin/RDS gene. *Retina,* 16**,** 405-10.

Namburi, P., Ratnapriya, R., Khateb, S., et al. 2016. Bi-allelic Truncating Mutations in CEP78, Encoding Centrosomal Protein 78, Cause Cone-Rod Degeneration with Sensorineural Hearing Loss. *American Journal of Human Genetics,* 99**,** 777-784.

Nikopoulos, K., Farinelli, P., Giangreco, B., et al. 2016. Mutations in CEP78 Cause Cone-Rod Dystrophy and Hearing Loss Associated with Primary-Cilia Defects. *American Journal of Human Genetics,* 99**,** 770-776.

Nishiguchi, K. M., Sokal, I., Yang, L., et al. 2004. A novel mutation (I143NT) in guanylate cyclase-activating protein 1 (GCAP1) associated with autosomal dominant cone degeneration. *Investigative Ophthalmology & Visual Science,* 45**,** 3863-3870.

Nong, E., Lee, W., Merriam, J. E., et al. 2014. Disease progression in autosomal dominant cone-rod dystrophy caused by a novel mutation (D100G) in the GUCA1A gene. *Documenta Ophthalmologica,* 128**,** 59-67.

Oishi, M., Oishi, A., Gotoh, N., et al. 2016. Next-generation sequencing-based comprehensive molecular analysis of 43 Japanese patients with cone and cone-rod dystrophies. *Molecular Vision,* 22**,** 150-160.

Ostergaard, E., Batbayli, M., Duno, M., et al. 2010. Mutations in PCDH21 cause autosomal recessive cone-rod dystrophy. *Journal of Medical Genetics,* 47**,** 665-669.

Paloma, E., Coco, R., Martínez-Mir, A., et al. 2002. Analysis of ABCA4 in mixed Spanish families segregating different retinal dystrophies. *Human Mutation,* 20**,** 476-476.

Paloma, E., Martínez-Mir, A., Vilageliu, L., et al. 2001. Spectrum of ABCA4 (ABCR) gene mutations in Spanish patients with autosomal recessive macular dystrophies. *Human Mutation,* 17**,** 504-510.

Papaioannou, M., Ocaka, L., Bessant, D., et al. 2000. An Analysis of ABCR Mutations in British Patients with Recessive Retinal Dystrophies. *Investigative Ophthalmology & Visual Science,* 41**,** 16-19.

Parry, D. A., Toomes, C., Bida, L., et al. 2009. Loss of the metalloprotease ADAM9 leads to cone-rod dystrophy in humans and retinal degeneration in mice. *American Journal of Human Genetics,* 84**,** 683-691.

Paunescu, K., Preising, M. N., Janke, B., et al. 2007. Genotype-Phenotype Correlation in a German Family with a Novel Complex CRX Mutation Extending the Open Reading Frame. *Ophthalmology,* 114**,** 1348-1357.e1.

Payne, A. M., Downes, S. M., Bessant, D. a. R., et al. 1998. A Mutation in Guanylate Cyclase Activator 1A (GUCA1A) in an Autosomal Dominant Cone Dystrophy Pedigree Mapping to a New Locus on Chromosome 6p21.1. *Human Molecular Genetics,* 7**,** 273-277.

Payne, A. M., Morris, A. G., Downes, S. M., et al. 2001. Clustering and frequency of mutations in the retinal guanylate cyclase (GUCY2D) gene in patients with dominant cone-rod dystrophies. *Journal of Medical Genetics,* 38**,** 611-614.

Piri, N., Gao, Y. Q., Danciger, M., et al. 2005. A substitution of G to C in the cone cGMP-phosphodiesterase gamma subunit gene found in a distinctive form of cone dystrophy. *Ophthalmology,* 112**,** 159-66.

Rahner, N., Nuernberg, G., Finis, D., et al. 2016. A novel C8orf37 splice mutation and genotype-phenotype correlation for cone-rod dystrophy. *Ophthalmic Genetics,* 37**,** 294-300.

Renner, A. B., Fiebig, B. S., Weber, B. H. F., et al. 2009. Phenotypic Variability and Long-term Follow-up of Patients With Known and Novel PRPH2/RDS Gene Mutations. *American Journal of Ophthalmology,* 147**,** 518-530.e1.

Riveiro-Alvarez, R., Lopez-Martinez, M.-A., Zernant, J., et al. 2013. Outcome of ABCA4 disease-associated alleles in autosomal recessive retinal dystrophies: retrospective analysis in 420 Spanish families. *Ophthalmology,* 120**,** 2332-2337.

Riveiro-Álvarez, R., Xie, Y. A., López-Martínez, M.-Á., et al. 2015. New mutations in the RAB28 gene in 2 Spanish families with cone-rod dystrophy. *JAMA Ophthalmology,* 133**,** 133-139.

Rivolta, C., Peck, N. E., Fulton, A. B., et al. 2001. Novel frameshift mutations in CRX associated with Leber congenital amaurosis. *Human Mutation,* 18**,** 550-551.

Roosing, S., Lamers, I. J. C., De Vrieze, E., et al. 2014. Disruption of the basal body protein POC1B results in autosomal-recessive cone-rod dystrophy. *American Journal of Human Genetics,* 95**,** 131-142.

Roosing, S., Rohrschneider, K., Beryozkin, A., et al. 2013. Mutations in RAB28, encoding a farnesylated small GTPase, are associated with autosomal-recessive cone-rod dystrophy. *American Journal of Human Genetics,* 93**,** 110-7.

Ruddle, J. B., Ebenezer, N. D., Kearns, L. S., et al. 2009. RPGR ORF15 genotype and clinical variability of retinal degeneration in an Australian population. *British Journal of Ophthalmology,* 93**,** 1151.

Salah, S. B., Kamei, S., Sénéćhal, A., et al. 2008. Novel KCNV2 Mutations in Cone Dystrophy with Supernormal Rod Electroretinogram. *American Journal of Ophthalmology,* 145**,** 1099-1106.

Sankila, E.-M., Joensuu, T. H., Hämäläinen, R. H., et al. 2000. A CRX mutation in a Finnish family with dominant cone-rod retinal dystrophy. *Human Mutation,* 16**,** 94-94.

Sergouniotis, P. I., Chakarova, C., Murphy, C., et al. 2014. Biallelic variants in TTLL5, encoding a tubulin glutamylase, cause retinal dystrophy. *American Journal of Human Genetics,* 94**,** 760-769.

Shaikh, R. S., Reuter, P., Sisk, R. A., et al. 2015. Homozygous missense variant in the human CNGA3 channel causes cone-rod dystrophy. *European Journal Of Human Genetics,* 23**,** 473-80.

Simonelli, F., Testa, F., Zernant, J., et al. 2004. Association of a Homozygous Nonsense Mutation in the ABCA4 (ABCR) Gene with Cone-Rod Dystrophy Phenotype in an Italian Family. *Ophthalmic Research,* 36**,** 82-88.

Singh, H. P., Jalali, S., Hejtmancik, J. F., et al. 2006. Homozygous Null Mutations in the ABCA4 Gene in Two Families With Autosomal Recessive Retinal Dystrophy. *American Journal of Ophthalmology,* 141**,** 906-913.

Small, K. W., Silva-Garcia, R., Udar, N., et al. 2008. New mutation, P575L, in the GUCY2D gene in a family with autosomal dominant progressive cone degeneration. *Archives of Ophthalmology,* 126**,** 397-403.

Smith, M., Whittock, N., Searle, A., et al. 2006. Phenotype of autosomal dominant cone–rod dystrophy due to the R838C mutation of the GUCY2D gene encoding retinal guanylate cyclase-1. *Eye,* 21**,** 1220.
Sohocki, M. M., Daiger, S. P., Bowne, S. J., et al. 2000a. Prevalence of mutations causing retinitis pigmentosa and other inherited retinopathies. *Human Mutation,* 17**,** 42-51.
Sohocki, M. M., Perrault, I., Leroy, B. P., et al. 2000b. Prevalence of AIPL1 Mutations in Inherited Retinal Degenerative Disease. *Molecular Genetics and Metabolism,* 70**,** 142-150.
Sohocki, M. M., Sullivan, L. S., Mintz-Hittner, H. A., et al. 1998. A Range of Clinical Phenotypes Associated with Mutations in CRX, a Photoreceptor Transcription-Factor Gene. *American Journal of Human Genetics,* 63**,** 1307-1315.
Sokal, I., Dupps, W. J., Grassi, M. A., et al. 2005. A novel GCAP1 missense mutation (L151F) in a large family with autosomal dominant cone-rod dystrophy (adCORD). *Investigative Ophthalmology & Visual Science,* 46**,** 1124-1132.
Stenirri, S., Battistella, S., Fermo, I., et al. 2006. De novo deletion removes a conserved motif in the C-terminus of ABCA4 and results in cone-rod dystrophy. *Clinical Chemistry and Laboratory Medicine (CCLM).*
Suga, A., Mizota, A., Kato, M., et al. 2016. Identification of Novel Mutations in the LRR-Cap Domain of C21orf2 in Japanese Patients With Retinitis Pigmentosa and Cone–Rod Dystrophy. *Investigative Ophthalmology & Visual Science,* 57**,** 4255-4263.
Swain, P. K., Chen, S., Wang, Q.-L., et al. 1997. Mutations in the Cone-Rod Homeobox Gene Are Associated with the Cone-Rod Dystrophy Photoreceptor Degeneration. *Neuron,* 19**,** 1329-1336.
Thiadens, A. A., Den Hollander, A. I., Roosing, S., et al. 2009. Homozygosity mapping reveals PDE6C mutations in patients with early-onset cone photoreceptor disorders. *American Journal of Human Genetics,* 85**,** 240-7.
Thiadens, A. A., Phan, T. M., Zekveld-Vroon, R. C., et al. 2012. Clinical course, genetic etiology, and visual outcome in cone and cone-rod dystrophy. *Ophthalmology,* 119**,** 819-26.
Thiadens, A. a. H. J., Roosing, S., Collin, R. W. J., et al. 2010. Comprehensive Analysis of the Achromatopsia Genes CNGA3 and CNGB3 in Progressive Cone Dystrophy. *Ophthalmology,* 117**,** 825-830.e1.
Thiagalingam, S., Mcgee, T. L., Weleber, R. G., et al. 2007. Novel Mutations in the KCNV2 Gene in Patients with Cone Dystrophy and a Supernormal Rod Electroretinogram. *Ophthalmic Genetics,* 28**,** 135-142.
Udar, N., Yelchits, S., Chalukya, M., et al. 2003. Identification of GUCY2D gene mutations in CORD5 families and evidence of incomplete penetrance. *Human Mutation,* 21**,** 170-171.
Van Ghelue, M., L. Eriksen, H., Ponjavic, V., et al. 2001. Autosomal dominant cone-rod dystrophy due to a missense mutation (R838C) in the guanylate cyclase 2D gene (GUCY2D) with preserved rod function in one branch of the family. *Ophthalmic Genetics,* 21**,** 197-209.
Wawrocka, A., Skorczyk-Werner, A., Wicher, K., et al. 2018. Novel variants identified with next-generation sequencing in Polish patients with cone-rod dystrophy. *Molecular Vision,* 24**,** 326-339.
Weigell-Weber, M., Fokstuen, S., Török, B., et al. 2000. Codons 837 and 838 in the retinal guanylate cyclase gene on chromosome 17p: Hot spots for mutations in autosomal dominant cone-rod dystrophy? *Archives of Ophthalmology,* 118**,** 300-300.
Wilkie, S. E., Li, Y., Deery, E. C., et al. 2001. Identification and functional consequences of a new mutation (E155G) in the gene for GCAP1 that causes autosomal dominant cone dystrophy. *American Journal of Human Genetics,* 69**,** 471-480.
Wissinger, B., Dangel, S., Jagle, H., et al. 2008. Cone dystrophy with supernormal rod response is strictly associated with mutations in KCNV2. *Investigative Ophthalmology & Visual Science,* 49**,** 751-7.
Wissinger, B., Gamer, D., Jägle, H., et al. 2001. CNGA3 mutations in hereditary cone photoreceptor disorders. *American Journal of Human Genetics,* 69**,** 722-737.

Wissinger, B., Schaich, S., Baumann, B., et al. 2011. Large deletions of the KCNV2 gene are common in patients with cone dystrophy with supernormal rod response. *Human Mutation,* 32**,** 1398-1406.

Wu, H., Cowing, J. A., Michaelides, M., et al. 2006. Mutations in the gene KCNV2 encoding a voltage-gated potassium channel subunit cause "cone dystrophy with supernormal rod electroretinogram" in humans. *American Journal of Human Genetics,* 79**,** 574-9.

Wycisk, K. A., Zeitz, C., Feil, S., et al. 2006. Mutation in the auxiliary calcium-channel subunit CACNA2D4 causes autosomal recessive cone dystrophy. *American Journal of Human Genetics,* 79**,** 973-977.

Xi, Q., Li, L., Traboulsi, E. I., et al. 2009. Novel ABCA4 compound heterozygous mutations cause severe progressive autosomal recessive cone-rod dystrophy presenting as Stargardt disease. *Molecular Vision,* 15**,** 638-645.

Xiao, X., Guo, X., Jia, X., et al. 2011. A recurrent mutation in GUCY2D associated with autosomal dominant cone dystrophy in a Chinese family. *Molecular Vision,* 17**,** 3271-3278.

Xu, F., Dong, F., Li, H., et al. 2013. Phenotypic characterization of a Chinese family with autosomal dominant cone–rod dystrophy related to GUCY2D. *Documenta Ophthalmologica,* 126**,** 233-240.

Yang, P., Chiang, P., Weleber, R. G., et al. 2015. Autosomal dominant retinal dystrophy with electronegative waveform associated with a novel RAX2 mutation. *JAMA Ophthalmology,* 133**,** 653-661.

Yang, Z., Chen, Y., Lillo, C., et al. 2008. Mutant prominin 1 found in patients with macular degeneration disrupts photoreceptor disk morphogenesis in mice. *J Clin Invest,* 118**,** 2908-16.

Yang, Z., Peachey, N., M Moshfeghi, D., et al. 2002. Mutations in the RPGR gene cause X-linked cone dystrophy. *Human Molecular Genetics,* 11**,** 605-11.

Yoshida, S., Yamaji, Y., Yoshida, A., et al. 2006. Novel triple missense mutations of GUCY2D gene in Japanese family with cone-rod dystrophy: Possible use of genotyping microarray. *Molecular Vision,* 12**,** 1558-64.

Yzer, S., Ingeborgh Van Den Born, L., Zonneveld, M., et al. 2007. Molecular and phenotypic analysis of a family with autosomal recessive cone-rod dystrophy and Stargardt disease. *Molecular Vision,* 13**,** 1568-72.

Zhao, X., Ren, Y., Zhang, X., et al. 2013. A novel GUCY2D mutation in a Chinese family with dominant cone dystrophy. *Molecular Vision,* 19**,** 1039-1046.
